# Influence of Doping and Excitation Powers on Optical Thermometry in Yb^3+^-Er^3+^ doped CaWO_4_

**DOI:** 10.1038/srep43383

**Published:** 2017-02-27

**Authors:** Xiangfu Wang, Yemin Wang, Yanyan Bu, Xiaohong Yan, Jing Wang, Peiqing Cai, Thiquynh Vu, Hyo Jin Seo

**Affiliations:** 1 College of Electronic Science and Engineering, Nanjing University of Posts and Telecommunications, Nanjing, 210046, People’s Republic of China; 2 Department of Physics and Interdisciplinary Program of Biomedical, Mechanical & Electrical Engineering, Pukyong National University, Busan 608-737, Republic of Korea

## Abstract

Optical thermometry has been widely studied to achieve an inaccessible temperature measurement in submicron scale and it has been reported that the temperature sensitivity depends mainly on host types. In this work, we propose a new method to improve the optical temperature sensitivity of Yb^3+^-Er^3+^ co-doped CaWO_4_ phosphors by doping with Li^+^, Sr^2+^, and Mg^2+^ ions and by controlling excitation powers of 980 nm laser. It is found that the thermometric parameters such as upconversion emission intensity, intensity ratio of green-to-red emission, fluorescence color, emission intensity ratios of thermally coupled levels (^2^H_11/2_/^4^S_3/2_), and relative and absolute temperature sensitivity can be effectively controlled by doping with Li^+^, Sr^2+^, and Mg^2+^ ions in the Yb^3+^-Er^3+^ co-doped CaWO_4_ system. Moreover, the relative sensitivity S_R_ and the absolute sensitivity S_A_ are proved to be dependent on the pump power of 980 nm laser. The sensitivities of S_R_ and S_A_ in Yb^3+^-Er^3+^ co-doped CaWO_4_ increase about 31.5% and 12%, respectively, by doping with 1 mol% Sr^2+^.

In recent years, more and more research works have been focused on detecting temperature in submicron scale through optical thermometry, due to wide application in nano-devices^1^,^2^,^3^. Optical thermometry is a method to calculate the temperature of phosphors by fitting the fluorescence intensity ratios (*FIR*) of thermally coupled levels at different temperate points^4^. Compared with conventional temperature measurement contact methods, the non-contact *FIR* technique overcomes some limitations of spatial resolution and accuracy of detection. The thermally coupled levels ^2^H_11/2_ and ^4^S_3/2_ of Er^3+^ ion were reported as one of preferable choice to achieve optical thermometry, due to their large energy gap and less overlap of two emission peaks between the ^2^H_11/2_ and ^4^S_3/2_ levels^1^,^5^,^6^. Three comprehensive reviews summarized the optical thermometry based on upconversion emissions of Er^3+^ doped phosphors, such as oxide crystals, glasses, core-shell heterojunction nanoparticles, and transparent glass ceramics containing fluoride nanocrystals^7^,^8^,^9^. It was reported that the optical temperature sensitivity of Er^3+^ doped phosphors depends mainly on host types irrespective of the other conditions, such as excitation powers, doping concentrations, and sizes and shapes of samples. The main weakness of the previous research works is the failure to address the question: If the power of excitation source changes from low to high, how the changes in power affect the optical temperature sensitivity? Rakov and Maciel observed the optical temperature sensitivity is inconsistent between continuous wave and pulsed (~5 ns) laser excitations in the Er^3+^-Yb^3+^ doped Y_2_SiO_5_ system^10^. Chen *et al*. reported that the intensity ratio of green and red emissions depends strongly on the excitation power density^11^. Thus, it is necessary to study the influence of pump power on optical temperature sensitivity.

If the excitation power is fixed, how to enhance the optical temperature sensitivity without changing the category of hosts? According to the crystal-field theory, the emissions from the ^2^H_11/2_ and ^4^S_3/2_ thermally coupled levels are inevitably affected by the crystal field around Er^3+^ ions in hosts^12^,^13^,^14^. In some works, the tuning of the upconversion emissions of Er^3+^ were realized by tailoring the crystal field around Er^3+^ ions. For examples, the enhancement of the upconversion luminescence was achieved in Yb^3+^/Er^3+^-codoped Y_2_O_3_ nano-crystals by tri-doping with Li^+^ ions^15^. Qin *et al*. reported and discussed a strategy to enhance upconversion emission in *β*-NaLuF_4_:Yb^3+^, Er^3+^ nano-crystals by tri-doping with La^3+^ ions^16^. The lattice distortion of hosts is induced by dopant ions with small ionic radius and affects strongly the crystal-field environment around Er^3+^ ions leading to increase in radiative transition probability from the excited state to the ground state of ^4^I_15/2_^17^. The doping of the ions with small ionic radius may be a preferable method to improve the optical temperature sensitivity.

Recently, the Yb^3+^-Er^3+^ co-doped CaWO_4_ phosphors were reported as one of excellent materials to achieve the optical thermometry based on the green emissions from the thermally coupled ^2^H_11/2_ and ^4^S_3/2_ levels of Er^3+^ ions^18^,^19^,^20^. The CaWO_4_ crystal has tetragonal structure with distorted [WO6] octahedral clusters containing two quadrilateral biconical units^21^. This asymmetric crystal structure can be efficiently distorted by doping with small ions giving rise to change in fluorescence intensity ratio of the ^2^H_11/2_ and ^4^S_3/2_ levels. It means that the optical temperature sensitivity can be improved by changing crystal-field environment around Er^3+^ ions. In this work, we study the influences of the tri-doping with Li^+^, Sr^2+^, and Mg^2+^ ions and excitation powers on optical thermometry in Yb^3+^-Er^3+^ co-doped CaWO_4_. The relative and absolute emission sensitivities can be controlled effectively by doping with Li^+^, Sr^2+^, and Mg^2+^ ions in Yb^3+^-Er^3+^ co-doped CaWO_4_ and by changing the pump power of 980 nm laser.

## Results

**Figure 1:**
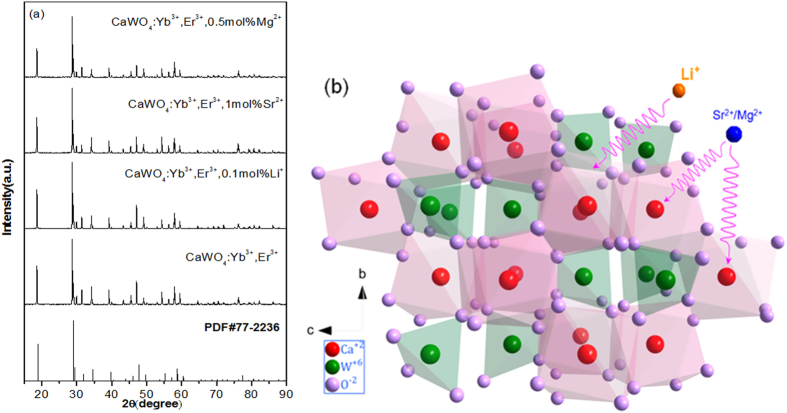
(**a**) XRD patterns of CaWO_4_ doped with Yb^3+^-Er^3+^, Yb^3+^-Er^3+^-0.1 mol%Li^+^, Yb^3+^-Er^3+^-1 mol%Sr^2+^, and Yb^3+^-Er^3+^-0.5 mol%Mg^2+^ and (**b**) the schematic views of unit cell of CaWO_4_ structure along a-direction.

Figure 1 presents the XRD patterns of as-synthesized CaWO_4_ doped with Yb^3+^-Er^3+^, Yb^3+^-Er^3+^-0.1%Li^+^, Yb^3+^-Er^3+^-1%Sr^2+^, and Yb^3+^-Er^3+^-0.5%Mg^2+^. The position and relative intensity of all the diffraction peaks can be readily indexed to pure Scheelite-type tetragonal CaWO_4_ according to the PDF card (No. 77-2236). The sharp and well defined diffraction peaks in the patterns suggest that the synthesized phosphors are well crystallized. The raw materials could not be detected in the as-synthesized samples confirming that Yb^3+^, Er^3+^, Li^+^, Sr^2+^ and Mg^2+^ are doped into the CaWO_4_ lattices. Figure 1(b) shows the crystal structure and atomic arrangement along the a-axis direction in CaWO_4_ which has tetragonal symmetry (ICSD 60550) with octahedral quadrilateral biconical crystals^21^. The tetragonal CaWO_4_ belongs the I41/aZ space group, and contains 4 formula units in each unit cell. In the unit cell of CaWO_4_, there are 4b Ca and 4a W cation sites. The Sr and Mg ions occupy the same 4b Ca cation sites, and the Li ion is located at an interstitial site in the CaWO_4_ lattice. In order to study the influence of the Li^+^, Sr^2+^ and Mg^2+^ doping on the morphology change, the scanning electron microscope (SEM) images are given in Figs S1, S2 and S3. One can find that the average size of Yb^3+^-Er^3+^ doped CaWO_4_ increases with the increase of Li^+^, Sr^2+^ and Mg^2+^ concentration, and its morphology becomes more irregular and agglomerated. The shape and size modifications are attributed to the surface charge redistribution of the crystal nucleus induced by inner electron charge transfer between the dopant ions and lattice cations^22^.

**Figure 2:**
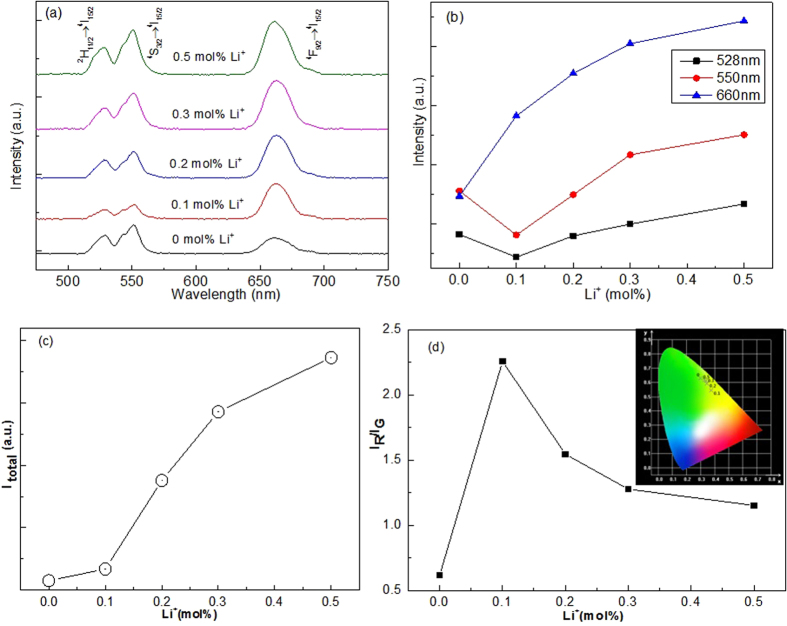
Li^+^ concentration dependent (**a**) upconversion spectra, (**b**) intensities of green and red emissions, (**c**) total emission intensity, and (**d**) the intensity ratios of the red to green emissions of CaWO_4_:Yb^3+^, Er^3+^, Li^+^.

The upconversion emission spectra of CaWO_4_:Yb^3+^, Er^3+^, Li^+^ excited by 980 nm infrared radiation are shown in Fig. 2(a). Three emission bands of Er^3+^ are observed at 528, 550, and 660 nm due to the ^2^H_11/2_ → ^4^I_15/2_, ^4^S_3/2_ → ^4^I_15/2_, and ^4^F_9/2_ → ^4^I_15/2_ transitions, respectively. No spectral shifts of the emission bands occur for various Li^+^ concentrations in CaWO_4_:Yb^3+^, Er^3+^, Li^+^. Compared with CaWO_4_:Yb^3+^, Er^3+^, the red emission intensity of CaWO_4_:Yb^3+^, Er^3+^, Li^+^ increases sharply with increasing Li^+^ concentration, while the green emission intensity changes irregularly with increasing Li^+^ concentration, as shown in Fig. 2(b). The total emission intensity increases sharply with increasing Li^+^ concentration from 0 to 0.5 mol%, as shown in Fig. 2(c). Figure 2(d) shows the intensity ratio of the red/green emissions (I_R_/I_G_) which is changed by changing Li^+^ concentration and the ratio reaches a maximum at 0.1 mol%. The CIE chromaticity in the inset of Fig. 2(d) shows that the emission color is tunable from greenish-yellow to green with increasing Li^+^ concentration. The spectrum modulation of CaWO_4_:Yb^3+^, Er^3+^ can also be achieved by doping with Sr^2+^ and Mg^2+^ ions, as shown in Figs S4 and S5. Similar to the Li^+^ ions doped in CaWO_4_:Yb^3+^, Er^3+^, the codoping with Sr^2+^ ions increases the total emission intensity of Er^3+^ ions (Fig. S4(c)). But for the Mg^2+^ ions, the total emission intensity of Er^3+^ ions rather decreases at higher concentration of 1.5 mol% (Fig. S5(c)). Consequently, the changes in total intensity and the intensity ratio of red to green emissions are not consistent in three kind of dopant ions (Li^+^, Sr^2+^ and Mg^2+^). Nevertheless, the results show that the spectrum modulation can be induced by doping of Li^+^, Sr^2+^ and Mg^2+^ as previously reported in Li^+^-doped Y_2_O_3_:Er^3+^ 15.

The enhancement of visible emissions induced by doping can be explained as follows: CaWO_4_ has octahedral quadrilateral biconical structure for which the Ca ion is coordinated with eight oxygen ions in the CaWO_4_ lattice, as shown in Fig. 1(b). Er^3+^ (eight coordination r = 1.004 Å) as an optical active center has a local structure with the ErO_8_ unit when the Er^3+^ is doped in CaWO_4_ lattice (eight coordination Ca^2+^, r = 1.12 Å). When the Li^+^ ions (eight coordination r = 0.92 Å) are doped into CaWO_4_:Er^3+^, they compensate the excess charge of 1^+^ between Er^3+^ and Ca^2+^. The Li^+^ ions may occupy the sites in or near the ErO_8_ units or diffuse to various interstitial sites in the CaWO_4_ lattice. The Sr^2+^ and Mg^2+^ ions (eight coordination r_Sr_ = 1.26 Å, r_Mg_ = 0.89 Å) substitute for the Ca^2+^ sites in CaWO_4_:Er^3+^ leading to the distortion of the Er-O bonds. As a result, the local symmetry of the crystal field around Er^3+^ lowers with the change of Coulomb interaction^17^. The forbidden intra-4*f* electronic transitions are partially allowed with increase of the intra-4*f* electronic transitions probability of the Er^3+^ ions^12^,^17^. Thus, the visible emissions can be enhanced by tailoring the local environments of the Er^3+^ ions by doping with Li^+^, Sr^2+^ and Mg^2+^ ions.

**Figure 3:**
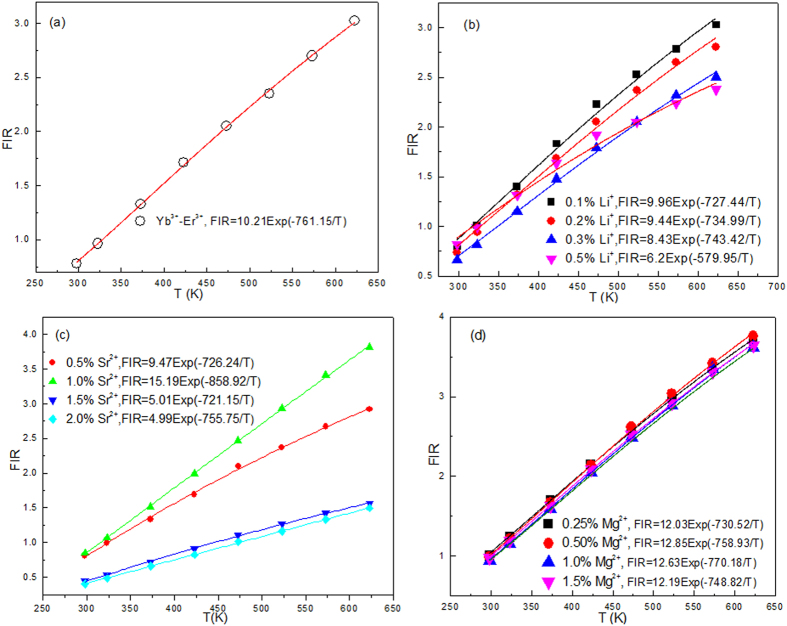
Temperature dependent FIR of 528 nm and 550 nm emissions of (**a**) Yb^3+^-Er^3+^co-doped CaWO_4_, (**b**) Yb^3+^-Er^3+^-Li^+^ tri-doped CaWO_4_, (**c**) Yb^3+^-Er^3+^-Sr^2+^ tri-doped CaWO_4_, and (**d**) Yb^3+^-Er^3+^-Mg^2+^ tri-doped CaWO_4_.

Based on upconversion emissions centered at 528 nm (^2^H_11/2_ → ^4^I_15/2_) and 550 nm (^4^S_3/2_ → ^4^I_15/2_) of Er^3+^ ions, the optical temperature sensing of Yb^3+^-Er^3+^ co-doped CaWO_4_ was studied by Li *et al*. by analyzing the temperature dependent fluoresce intensity ratio (FIR)^19^. Figure 3 shows the influence of doping with Li^+^, Sr^2+^, and Mg^2+^ on optical temperature sensing of Yb^3+^-Er^3+^ doped CaWO_4_. According to previous works^4^,^19^, the *FIR* of two thermally coupled levels of ^2^H_11/2_ and ^4^S_3/2_ can be fitted by the Boltzmann distributing law


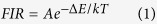


where *A* is a constant, Δ*E* is the energy difference between thermally coupled levels, *k* is the Boltzmann constant, and *T* is the absolute temperature. The relative sensitivity *S*_*R*_ and the absolute sensitivity *S*_*A*_ are defined as


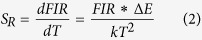



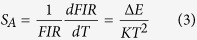


**Figure 4:**
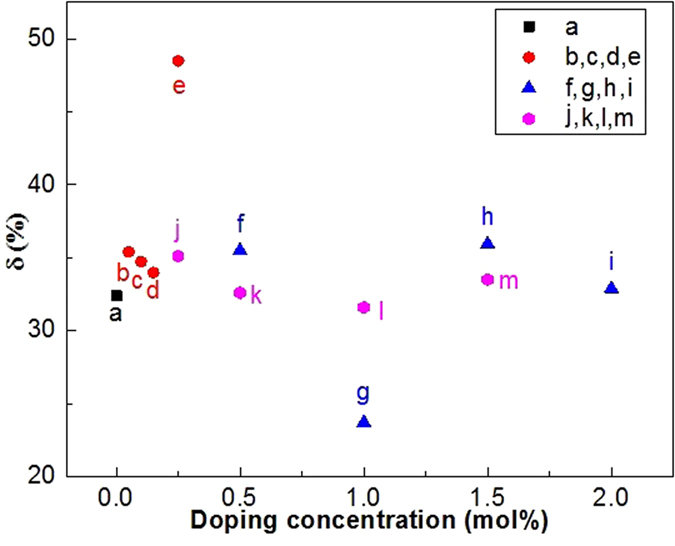
Doping dependent *δ* values in (a) Yb^3+^-Er^3+^ doped CaWO_4_, (b, c, d, e) Yb^3+^-Er^3+^-xLi^+^ (x = 0.1, 0.2, 0.3, and 0.5 mol%) doped CaWO_4_, (f,g,h,i) Yb^3+^-Er^3+^-ySr^2+^ (y = 0.5, 1.0, 1.5 and 2.0 mol%) doped CaWO_4_, and (j,k,l,m) Yb^3+^-Er^3+^-zMg^2+^ (z = 0.25, 0.5, 1.0, and 1.5 mol%) doped CaWO_4_.

Figure 3 shows the FIR as a function of temperature for various doping systems. The solid lines in Fig. 3 are best fit results obtained using Eq. (1). All the data are well fitted to Eq. (1). It is obvious that the fit results depend strongly on the types of dopant ions. Compared with the fit result for Yb^3+^-Er^3+^ co-doped CaWO_4_, the fit results vary much more for Yb^3+^-Er^3+^-M^n+^ (M^n+^ = Li^+^, Sr^2+^, and Mg^2+^) tri-doped CaWO_4_. The values of *ΔE* can be calculated from the data points of luminescence intensity ratios at different temperatures using Eq. (1). This indicates that the different types of dopant ions cause different values of *ΔE*. The peaks of green emissions at 528 and 550 nm do not shift with temperature for all the samples. However, the experimental value of *ΔE* should be unique for thermometry. As observed in Fig. 4 the changes in error also occur between the best-fit and experimental values of *ΔE* depending on the types of dopants and their concentrations. The error *δ* between *ΔE*_*f*_ and *ΔE*_*m*_ is expressed as:


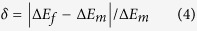


where *ΔE*_*f*_ is the fit value of *ΔE*, and *ΔE*_*m*_ is experimental value of *ΔE* from the spectrum^9^. The experimental value of *ΔE* is 757.6 cm^−1^ from the spectra in Fig. 2. The errors *δ* for Yb^3+^-Er^3+^ co-doped and Yb^3+^-Er^3+^-M^n+^ (M^n+^ = Li^+^, Sr^2+^, and Mg^2+^) tri-doped CaWO_4_ are calculated using Eq. (4) and the results are shown in Fig. 4. One can find that the values of error *δ* depend strongly on the types of dopant ions. All the values of error *δ* are more than 20%, and are too large to be ignored. Moreover, the value of error *δ* for Yb^3+^-Er^3+^ co-doped CaWO_4_ exceeds 30%. The large values of δ are attributed to the inconsistency between the restriction of Eq. (1) and luminescence mechanism at high temperature. As a theoretical model, Eq. (1) was proposed only by considering the Boltzmann distribution and the radiative transitions in the thermally coupled levels^23^. The temperature dependent nonradiative relaxation and energy transfer between the host and the rare-earth ions were not be considered at all. In fact, the nonradiative relaxation and energy transfer become active at high temperature^24^ giving rise to the large errors of *ΔE* in the optical temperature sensing process. The large errors of *ΔE* were also observed in more than fifty kinds of materials^9^ as well as Yb^3+^-Er^3+^ co-doped CaWO. This means that the *ΔE* is an unreasonable parameter to evaluate the optical temperature sensitivity S_R_ and S_A_. Thus, Eqs (2) and (3) should be modified to be reasonable for optical thermometry.

**Figure 5:**
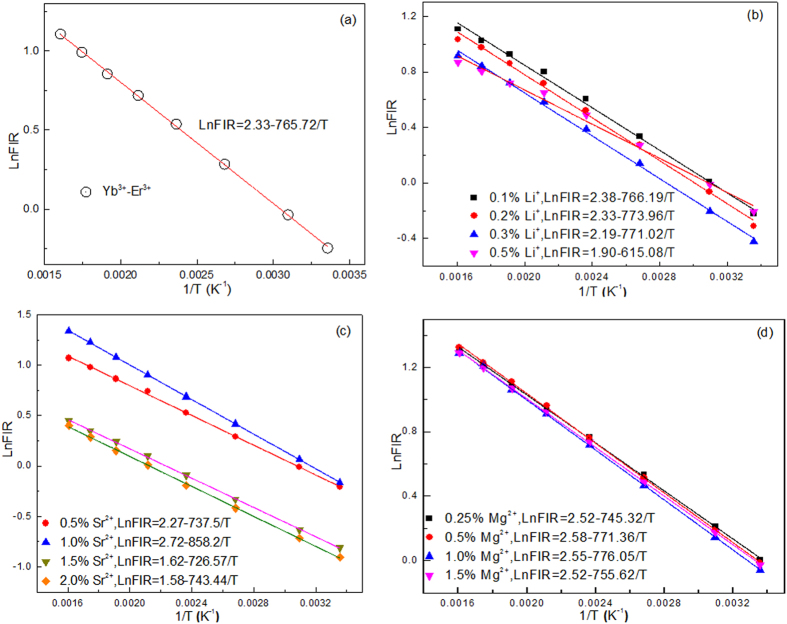
Temperature dependent relationship between the LnFIR and 1/T of (**a**) Yb^3+^-Er^3+^ doped CaWO_4_, (**b**) Yb^3+^-Er^3+^-Li^+^ doped CaWO_4_, (**c**) Yb^3+^-Er^3+^-Sr^2+^ doped CaWO_4_, and (**d**) Yb^3+^-Er^3+^-Mg^2+^ doped CaWO_4_.

**Figure 6:**
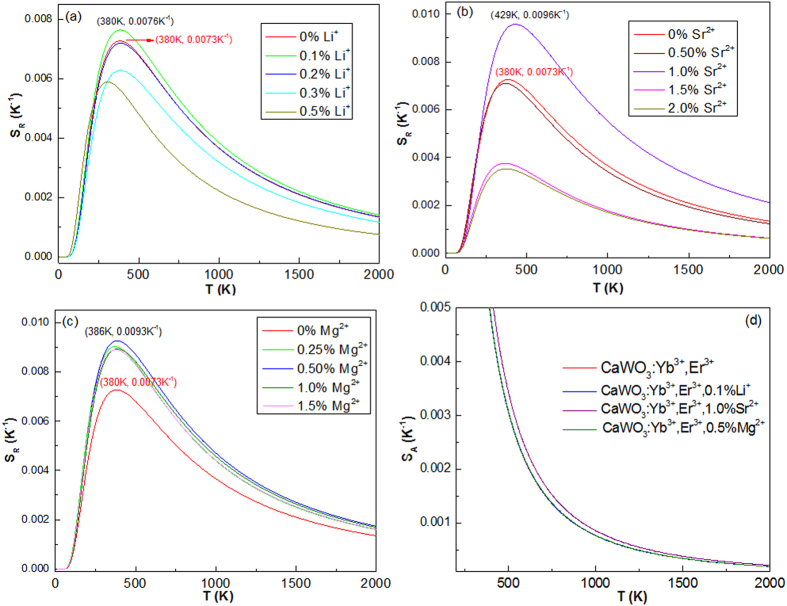
Optical temperature sensitivities *S*_*R*_ and *S*_*A*_ as a function of temperature for various dopant ions. (**a**) Yb^3+^-Er^3+^-Li^+^, (**b**) Yb^3+^-Er^3+^-Sr^2+^, and (**c**) Yb^3+^-Er^3+^-Mg^2+^ doped CaWO_4_. (**d**) Temperature dependent *S*_*A*_.

Considering the nonradiative relaxation and energy transfer at high temperature, Eq. (1) is modified as


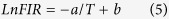


where *a* is constant dependent on the materials^10^. The *b* is a correction term for the comprehensive population of thermally coupled energy levels induced by not only the thermal population but also the nonradiative relaxation and energy transfer. The relative sensitivity *S*_*R*_ and the absolute sensitivity *S*_*A*_ are defined as


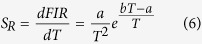



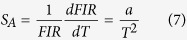


where a and b are from Eq. (5). Figure 5 shows the temperature dependent *FIR* of 528 and 550 nm green emissions in CaWO_4_ doped with different types of dopant ions. One can find that the experimental points can be fitted well with a linear mode. The slopes of the fit results depend on the types of dopant ions and concentrations. It means that the *FIR* of thermally coupled levels (^2^H_11/2_/^4^S_3/2_) are susceptible to the ion doping. Notably, all the *a* values in Fig. 5 are not consistent with those values of *ΔE/k* from Eq. (1) as shown in Fig. 3. In general, no difference in the *a* value is found between in Eqs (1) and (5), if considering only the thermal population. This deviation proves that it is unreasonable to use the *ΔE* to evaluate the optical temperature sensitivities *S*_*R*_ and *S*_*A*_. Figure 6 shows temperature dependent sensitivities *S*_*R*_ and *S*_*A*_ of CaWO_4_ doped with different types of dopant ions. All the sensitivity curves exhibit maxima with increasing temperature. The maximum *S*_*R*_ value of all the tri-doped CaWO_4_ systems changes non-monotonically with increasing dopant concentration, but the maximum S_*R*_ value of the Yb^3+^-Er^3+^ co-doped CaWO4 system are enhanced obviously by doping with Li^+^, Sr^2+^ and Mg^2+^ ions in optimum dopant concentrations. The maximum *S*_*R*_ value of Yb^3+^-Er^3+^ co-doped CaWO_4_ is estimated to be 0.0073 K^−1^ at 380 K, while 0.0076 K^−1^ at 380 K for Yb^3+^-Er^3+^-Li^+^ (0.1 mol%), 0.0096 K^−1^ at 429 K for Yb^3+^-Er^3+^-Sr^2+^ (1.0 mol%), and 0.0093 K^−1^ at 386 K for Yb^3+^-Er^3+^-Mg^2+^ (0.5 mol%) systems. The corresponding *S*_*A*_ values decrease with increasing temperature as shown in Fig. 6(d). The *S*_*A*_ value of Yb^3+^-Er^3+^ co-doped CaWO_4_ is enhanced by doping with Sr^2+^ ions. The values of *S*_*R*_ and *S*_*A*_ indicate that the resulting Yb^3+^-Er^3+^-Sr^2+^ tri-doped CaWO_4_ might be a promising candidate for optical temperature sensors.

The thermal stability of emission bands plays a key role in the optical temperature sensors. In order to explore the thermal stability, the temperature dependent log–log plots of emission intensity and pump power need to be investigated. The upconversion emission intensity *I* and excitation power *P* is expressed as follows


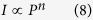


where n is the number of photons absorbed to pump the population in particular level in upconversion process^13^. Theoretically, the n values of green and red emissions of Er^3+^ ions excited by 980 nm are close to 2^24^. The temperature dependent log–log plots of emission intensity and pump power for green and red emissions are shown in Fig. S6. The slopes of the fit results for green and red emissions of Yb^3+^-Er^3+^ co-doped CaWO_4_ depend on temperature, and show that the upconversion process involves two photons to contribute green and red emissions. The slopes of the red emissions are much smaller than 2, which means that the red emission is not stable at high temperature^24^. Compared with the slopes of Yb^3+^-Er^3+^ co-doped CaWO_4_, those of the best-fit results for green and red emissions of Yb^3+^-Er^3+^-1.0%Sr^2+^ tri-doped CaWO_4_ are closer to 2. It means that Yb^3+^-Er^3+^-1.0%Sr^2+^ tri-doped CaWO_4_ has high thermal stability in emission bands than Yb^3+^-Er^3+^ co-doped CaWO_4_.

**Figure 7:**
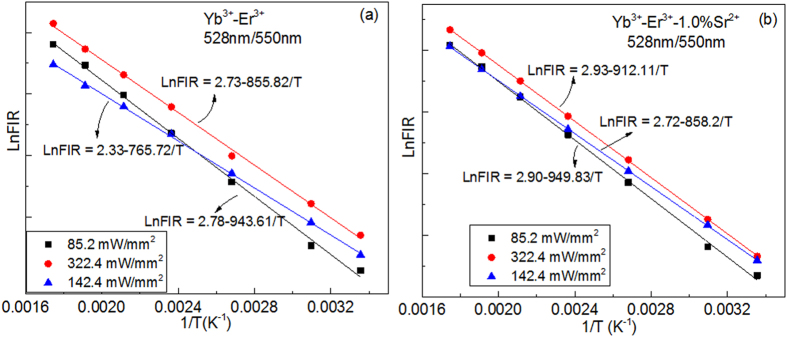
The LnFIR as a function of 1/T for various excitation powers for (**a**) Yb^3+^-Er^3+^ doped CaWO_4_ and (**b**) Yb^3+^-Er^3+^-1.0%Sr^2+^ doped CaWO_4_.

**Figure 8:**
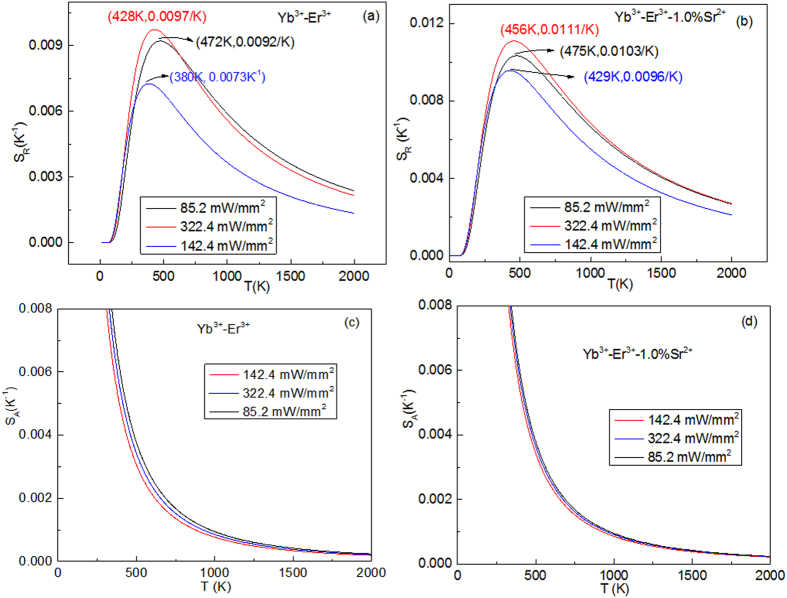
Excitation powers dependent S_A_ and S_R_ of Yb^3+^-Er^3+^ doped CaWO_4_ and Yb^3+^-Er^3+^-1.0%Sr^2+^ doped CaWO_4_.

Together with the thermal stability of emission bands, the excitation power dependence of the luminescence spectra was investigated in the temperature range from 298 to 573 K by using a 980 nm laser with pump powers of 85.2 and 322.4 mW/mm^2^, as shown in Fig. S7. The luminescence spectra contain green and red emission bands, which are assigned to the ^4^I_15/2_ → ^2^H_11/2_ (528 nm), ^4^I_15/2_ → ^4^S_3/2_ (550 nm), and ^4^I_15/2_ → ^4^F_9/2_ (660 nm) transitions of Er^3+^ ion. One can find that the intensities of 550 and 660 nm emission bands greatly decrease as temperature increases, while the intensity of 528 nm emission bands greatly increase as temperature increases. No peak shift of the emission bands induced by the doping and excitation powers is observed. The Ln*FIR* as a function of 1/T for various excitation powers are illustrated in Fig. 7. The slopes of the fit results at low excitation powers are different from those at high excitation powers. Moreover, the slopes of Yb^3+^-Er^3+^ co-doped CaWO_4_ are different from those of Yb^3+^-Er^3+^-1%Sr^2+^ tri-doped CaWO_4_ at the same excitation power. It means that the *FIR* of thermally coupled levels (^2^H_11/2_/^4^S_3/2_) are also susceptible to excitation powers like the dopant ions described above. The excitation power dependences of S_A_ and S_R_ are illustrated in Fig. 8. One can find that the values of S_A_ and S_R_ change non-monotonically with increasing excitation powers. The large value of S_A_ and S_R_ appear at the excitation power of 322.4 mW/mm^2^. The values of S_A_ and S_R_ of Yb^3+^-Er^3+^ co-doped CaWO_4_ are more susceptible to excitation powers than those of Yb^3+^-Er^3+^-1%Sr^2+^ tri-doped CaWO_4_. Thus, the Yb^3+^-Er^3+^-1%Sr^2+^ tri-doped CaWO_4_ is a better candidate for optical temperature sensors than Yb^3+^-Er^3+^ co-doped CaWO_4_ by considering the stabilities induced by temperature and excitation powers.

## Conclusions

In this work, a series of Yb^3+^-Er^3+^ co-doped and Yb^3+^-Er^3+^-M^n+^ (M^n+^ = Li^+^, Sr^2+^, and Mg^2+^) tri-doped CaWO_4_ phosphors were synthesized by using the high-temperature solid-state reaction method. The structural property of resulting powder is investigated by the X-ray diffraction. The upconversion emission intensity, green-to-red emission intensity ratio, fluorescence color of the Yb^3+^/Er^3+^ co-doped sample are controlled by doping Li^+^, Sr^2+^ and Mg^2+^ ions. The fluorescence intensity ratios of thermally coupled levels (^2^H_11/2_/^4^S_3/2_) and optical temperature sensitivity are observed to be dependent on dopant ions and excitation powers. It is found that the temperature sensitivity of CaWO_4_:5%Yb^3+^, 0.3%Er^3+^, 1.0%Sr^2+^ can reach a maximum relative sensitivity of 0.0111 K^−1^ at 456 K and absolute sensitivity of 949.8/T^2^, which is much higher than the reported temperature sensors based on Er^3+^ green luminescence. This work opens a new method to increase the optical temperature sensitivity of rare earth ions doped materials.

## Methods

All starting materials are CaCO_3_ (AR), WO_3_ (99.99%), Er_2_O_3_ (99.99%), Yb_2_O_3_ (99.99%), Li_2_CO_3_ (AR), SrO (AR) and MgO (AR). All the chemicals were used as received without any further purification.

The solid-state reaction method is used to synthesize CaWO_4_:Er^3+^/Yb^3+^ and CaWO_4_:Er^3+^/Yb^3+^/M^n+^ (M^n+^ = Li^+^, Sr^2+^ and Mg^2+^) phosphors. The molar ratio of CaWO_4_:Er^3+^/Yb^3+^ sample is as follows: 48.7CaCO_3_-48.7WO_3_-0.15Er_2_O_3_-2.45Yb_2_O_3_. The molar ratios of CaWO_4_:Er^3+^/Yb^3+^/Li^+^ samples are as follows: 48.7CaCO_3_-48.7WO_3_-0.15Er_2_O_3_-2.45Yb_2_O_3_-xLi_2_CO_3_, x = 0.05, 0.10, 0.15, and 0.25; The molar ratios of CaWO_4_:Er^3+^/Yb^3+^/Sr^2+^ samples are as follows: (48.7-y)CaCO_3_-48.7WO_3_-0.15Er_2_O_3_-2.45Yb_2_O_3_-ySrO, y = 0.50, 1.00, 1.50 and 2.00. The molar ratios of CaWO_4_: Er^3+^/Yb^3+^/Ca^2+^ samples are as follows: (48.7-z)CaCO_3_-48.7WO_3_-0.15Er_2_O_3_-2.45Yb_2_O_3_-zMgO, z = 0.25, 0.50, 1.00, and 1.50. The starting materials are entirely mixed and ground with alcohol for 1 hour. The powder is subsequently sintered in a furnace for 10 h at 1350 °C. Finally, a series of the required samples in the form of white powder are obtained after cooling down naturally.

Structures of the samples were investigated by X-ray diffraction (XRD) using a X’TRA (Switzerland ARL) equipment provided with Cu tube with K_α_ radiation at 1.54056 Å. The size and shape of the samples were observed using a scanning electron microscope (LEO-1550). Luminescence spectra were obtained by the Acton SpectraPro Sp-2300 Spectrophotometer with a photomultiplier tube equipped with 980 nm laser as the excitation sources. Different temperature spectra were obtained by using an INSTEC HCS302 Hot and Cold System.

## Additional Information

**How to cite this article:** Wang, X. *et al*. Influence of Doping and Excitation Powers on Optical Thermometry in Yb^3+^-Er^3+^ doped CaWO_4_. *Sci. Rep.* **7**, 43383; doi: 10.1038/srep43383 (2017).

**Publisher's note:** Springer Nature remains neutral with regard to jurisdictional claims in published maps and institutional affiliations.

## Supplementary Material

Supplementary Information
