# Supplementary material for: Influence of Doping and Excitation Powers on Optical Thermometry in Yb3+-Er3+ doped CaWO4
Source: Sci Rep. 2017 Feb 27;7:43383. doi: 10.1038/srep43383 (PMC5327477; doi:10.1038/srep43383)
Supplement: Supplementary Information [file srep43383-s1.doc]

Supplementary Information

Influence of Doping and Excitation Powers on Optical Thermometry in Yb^3+^-Er^3+^doped CaWO_4_

**Xiangfu Wang^1,2^, Yemin Wang^1^, Yanyan Bu^1^, Xiaohong Yan^1^, Jing Wang,^2^**

**Peiqing Cai,^2^ Thiquynh Vu,^2^ and Hyo Jin Seo^2*^**

**^1^College of Electronic Science and Engineering, Nanjing University of Posts and Telecommunications, Nanjing, 210046, People’s Republic of China, ^2^Department of Physics and Interdisciplinary Program of Biomedical, Mechanical & Electrical Engineering,** **Pukyong National University, Busan 608-737, Republic of Korea.**

**Correspondence and requests for materials should be addressed to H.S. (email: hjseo@pknu.ac.kr)**

**
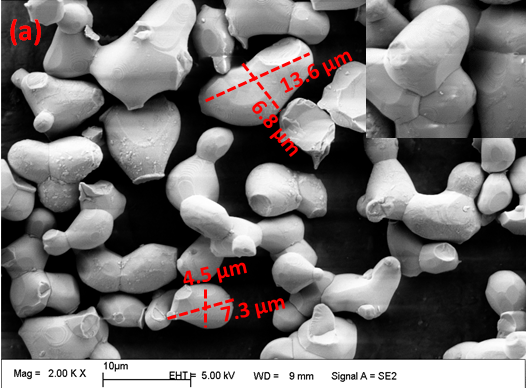
**

**
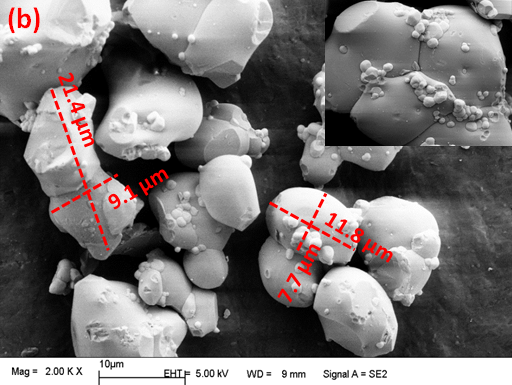
**

**
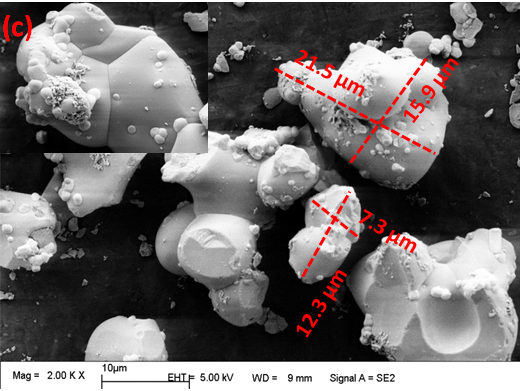
**

Figure S1. SEM images of (a)Yb^3+^-Er^3+^ doped CaWO_4_,(b)Yb^3+^-Er^3+^-0.1mol%Li^+^ doped CaWO_4_,(c)Yb^3+^-Er^3+^ -0.5mol%Li^+^ doped CaWO_4_. The inserts are magnified images.

**
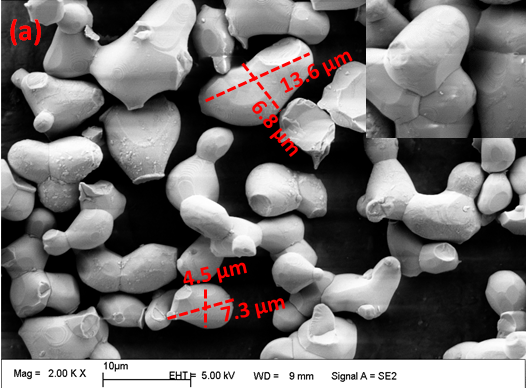
**

**
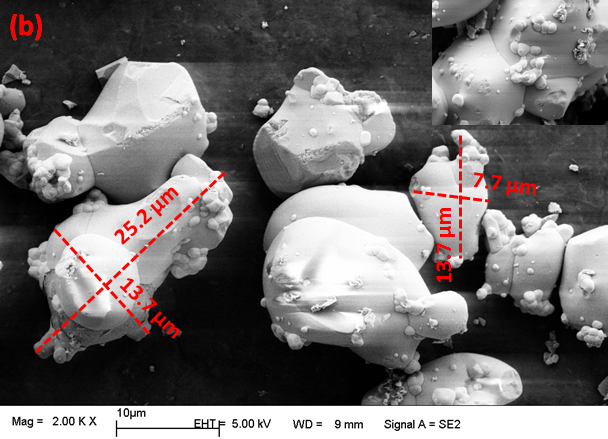
**

**
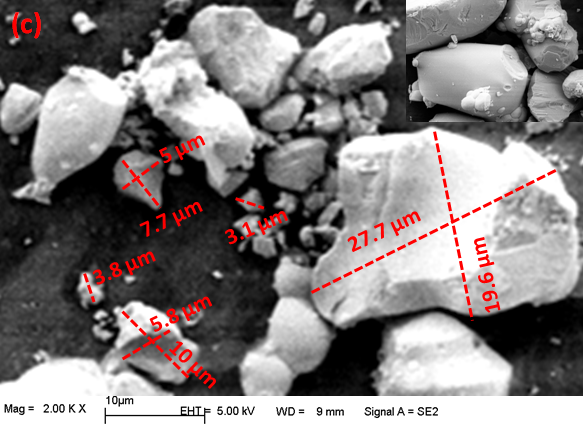
**

Figure S2. SEM images of (a)Yb^3+^-Er^3+^ doped CaWO_4_,(b)Yb^3+^-Er^3+^-1mol%Sr^2+^ doped CaWO_4_, (c)Yb^3+^-Er^3+^- 2mol%Sr^2+^ doped CaWO_4_.The inserts are magnified images.

**
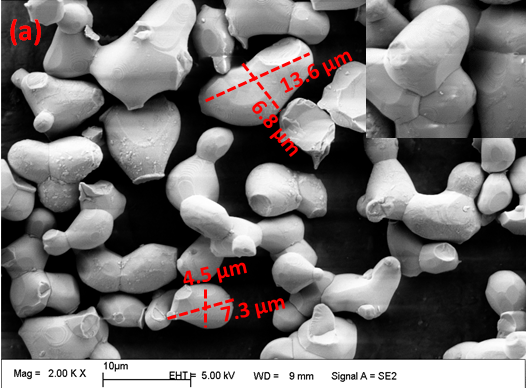
**

**
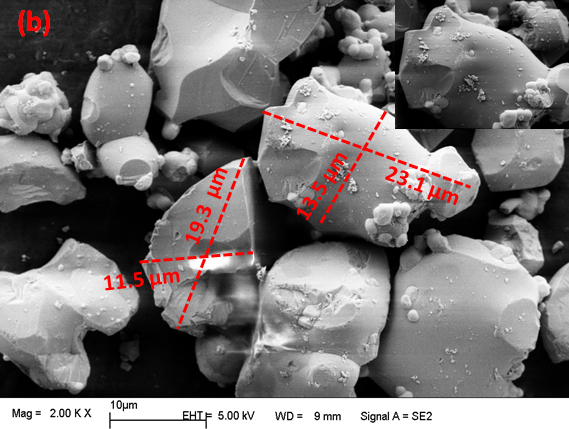
**

**
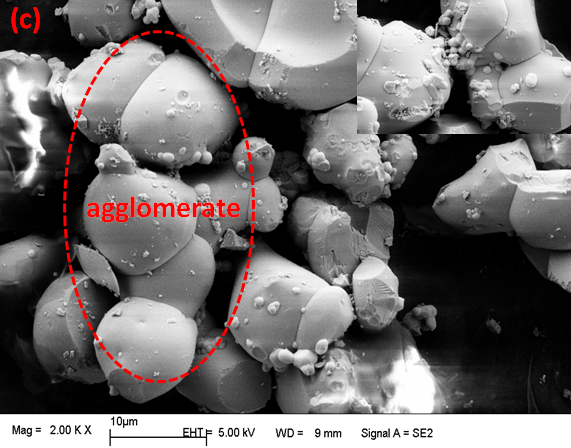
**

Figure S3. SEM images of (a)Yb^3+^-Er^3+^ doped CaWO_4_,(b)Yb^3+^-Er^3+^-1mol%Mg^2+^ doped CaWO_4_, (c)Yb^3+^- Er^3+^- 1.5mol%Mg^2+^ doped CaWO_4_. The inserts are magnified images.


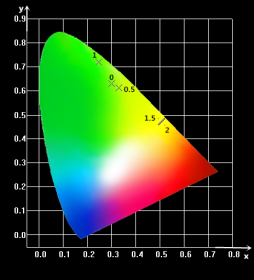
Figure S4. Sr^2+^ concentration dependent (a) upconversion spectra, (b) intensities of green and red emissions, (c) total emission intensity, and (d) the intensity ratios of the red to green emissions of CaWO_4_:Yb^3+^,Er^3+^,Sr^2+^.


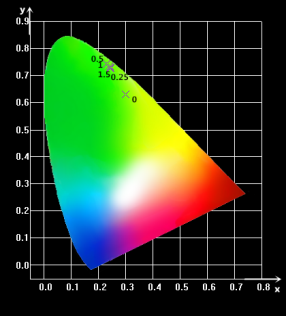


Figure S5. Mg^2+^ concentration dependent (a) upconversion spectra, (b) intensities of green and red emissions, (c) total emission intensity, and (d) the intensity ratios of the red to green emissions of CaWO_4_:Yb^3+^,Er^3+^,Mg^2+^.

Figure S6. Temperature dependent log–log plots of intensity and pumping powers for emissions of (a) 550 nm from Yb^3+^-Er^3+^ co-doped CaWO_4_, (b) 660 nm from Yb^3+^-Er^3+^ co-doped CaWO_4_, (c) 550 nm from Yb^3+^-Er^3+^-1%Sr^2+^ tri-doped CaWO_4_, and (d) 660 nm from Yb^3+^-Er^3+^-1%Sr^2+^ tri-doped CaWO_4_.

Figure S7． Temperature dependent photoluminescence spectra of Yb^3+^-Er^3+^ co-doped CaWO_4_ and Yb^3+^-Er^3+^-1%Sr^2+^ tri-doped CaWO_4_ at low 85.2 mW/mm^2^ excitation power and at high 322.4 mW/mm^2^ excitation power.
